# Weight Loss That Lasts: Reviewing the Long-Term Impact of GLP-1 Receptor Agonists

**DOI:** 10.7759/cureus.88334

**Published:** 2025-07-19

**Authors:** Md Yasir Shah, Ahmad Mohammad, Rabia Bashir Ahmed Samejo, Siddharth Rana, Shivam Singla, Raja Irsalan Aurangzeb, Muhammad M Tariq, Muhammad Hamza Zamir, Umer Farooq, Raja Faizan Aurangzeb, Bhavna Singla, Abdur Rehman

**Affiliations:** 1 Internal Medicine, Dhaka National Medical Institute Hospital, Dhaka, BGD; 2 Internal Medicine, Hurley Medical Center, Flint, USA; 3 Internal Medicine, Shaheed Mohtarma Benazir Bhutto Medical University, Larkana, PAK; 4 Internal Medicine, Jhalawar Medical College, Jhalawar, IND; 5 Internal Medicine, TidalHealth Peninsula Regional, Salisbury, USA; 6 Internal Medicine, Fauji Foundation Hospital Rawalpindi, Rawalpindi, PAK; 7 Internal Medicine, Foundation University Medical College, Islamabad, PAK; 8 Internal Medicine, Erie County Medical Center, Buffalo, USA; 9 Medicine and Surgery, King Edward Medical University, Lahore, PAK

**Keywords:** exenatide, glp-1 receptor agonists, liraglutide, long-term treatment, obesity management, randomized controlled trials, safety profile, semaglutide, sustained weight loss, tirzepatide

## Abstract

This systematic review evaluates the long-term efficacy and safety of GLP-1 receptor agonists in the management of obesity, focusing on data from high-quality randomized controlled trials published between 2018 and 2025. This review synthesizes findings from studies assessing agents such as semaglutide, liraglutide, tirzepatide, and exenatide in diverse populations, including adults with and without type 2 diabetes and adolescents with severe obesity. Most studies demonstrated sustained weight loss and favorable glycemic control over treatment durations of 40-120 weeks. Additionally, the agents showed generally acceptable safety profiles, with gastrointestinal side effects being the most frequently reported adverse events. The results reinforce the need to conceptualize obesity as a chronic condition requiring long-term pharmacological management. Despite the promising outcomes, limitations in follow-up durations, population diversity, and real-world generalizability highlight areas for future research.

## Introduction and background

Obesity has emerged as a global epidemic, with the World Health Organization estimating that over one billion people worldwide are currently classified as overweight or obese. It is a chronic, relapsing disease associated with numerous comorbidities, including type 2 diabetes mellitus (T2DM), cardiovascular disease, obstructive sleep apnea, and various cancers [1]. Traditional management strategies such as lifestyle modification and behavioral therapy often lead to modest weight loss, but maintaining these results long-term remains a significant challenge due to physiological adaptations that favor weight regain [2].

Pharmacological interventions have become a crucial component in the management of obesity, especially for individuals who do not respond adequately to lifestyle changes alone [3]. Among these, glucagon-like peptide-1 receptor agonists (GLP-1 RAs) have gained considerable attention for their dual benefits in glycemic control and weight reduction. Initially approved for T2DM, agents such as liraglutide and semaglutide have shown substantial efficacy in reducing body weight and improving cardiometabolic parameters even in non-diabetic populations [4,5]. Their mechanisms - slowing gastric emptying, increasing satiety, and reducing appetite - have made them highly effective options for obesity treatment.

While multiple randomized controlled trials (RCTs) have confirmed the short-term efficacy and safety of GLP-1 RAs, the long-term outcomes, particularly regarding sustained weight loss, weight regain, and safety profiles, remain areas of active investigation [6]. Understanding how well these agents maintain weight loss over time and how tolerable they remain during extended use is vital for clinical decision-making and guideline development. Additionally, as newer agents like tirzepatide enter clinical practice, evaluating their comparative durability becomes increasingly relevant. Therefore, the objective of this systematic review was to critically evaluate the sustained efficacy and safety of GLP-1 receptor agonists in the long-term management of obesity, focusing on randomized controlled trials that assess weight maintenance, cardiometabolic outcomes, and adverse event profiles.

## Review

Materials and methods

Study Design

This study was conducted as a systematic review following the Preferred Reporting Items for Systematic Reviews and Meta-Analyses (PRISMA) 2020 guidelines to ensure methodological transparency and replicability [7]. The aim was to evaluate the sustained efficacy and safety of GLP-1 receptor agonists in the long-term management of obesity.

Eligibility Criteria and PICO Framework

The inclusion criteria were structured using the PICO framework [8]. The population of interest consisted of adolescents or adults diagnosed with obesity or overweight, with or without associated comorbidities such as cardiovascular disease or type 2 diabetes mellitus. The interventions reviewed were long-term therapies using GLP-1 receptor agonists, including semaglutide, liraglutide, tirzepatide, and exenatide, administered for a minimum of 40 weeks. Comparators included placebo, standard care, or other pharmacological agents. Primary outcomes included sustained weight loss, maintenance of glycemic control, and safety indicators such as adverse events, discontinuation rates, and tolerability profiles. Studies were included if they were randomized controlled trials (RCTs), post hoc analyses of RCTs, or well-designed multicenter trials. Exclusion criteria included studies with a duration of less than 40 weeks, those without obesity-specific outcomes, animal or in vitro studies, and studies lacking relevant comparator arms.

Search Strategy

A systematic search was performed across multiple databases, including PubMed, Cochrane CENTRAL, Embase, and ClinicalTrials.gov, for articles published between January 2018 and April 2025. The search strategy included a combination of Medical Subject Headings (MeSH) and free-text terms related to GLP-1 receptor agonists (e.g., “semaglutide,” “liraglutide,” and “GLP-1 agonist”), obesity management, long-term efficacy, weight maintenance, and safety outcomes. The search was refined iteratively to narrow down results to trials that explicitly reported data beyond the acute treatment phase, focusing on sustained outcomes.

Study Selection and Screening

Two independent reviewers screened all titles and abstracts retrieved from the search. Full-text articles were obtained for studies meeting the initial eligibility criteria. Discrepancies during selection were resolved through discussion and, when necessary, consultation with a third reviewer. A final total of six studies were selected after applying the inclusion and exclusion criteria. Reasons for exclusion were documented at each stage in accordance with the PRISMA flow diagram.

Data Extraction

For each included study, detailed data were extracted into a predesigned table. This included study identifiers (author, year), population characteristics (age, BMI, comorbidities), intervention and comparator regimens, duration of follow-up, and primary outcomes related to efficacy and safety. Secondary outcomes such as behavioral or metabolic parameters were also noted. Extraction was performed independently by two reviewers and cross-checked for accuracy and completeness.

Risk of Bias Assessment

To assess methodological quality and internal validity, we applied the Cochrane Risk of Bias 2.0 (RoB 2) tool for randomized controlled trials [9]. Each study was evaluated across five domains as follows: bias arising from the randomization process, deviations from intended interventions, missing outcome data, measurement of the outcome, and selection of the reported result. Ratings of low, some concerns, or high risk were assigned to each domain and then synthesized into an overall judgment of risk of bias. Assessments were done independently by two reviewers, with consensus reached in cases of disagreement.

Data Synthesis

Given the heterogeneity in outcome measures, follow-up durations, and intervention protocols, a qualitative synthesis approach was adopted instead of meta-analysis. Key findings across trials were narratively synthesized to identify trends in sustained weight loss, cardiometabolic effects, adverse events, and treatment discontinuation. Differences between agents and populations were specifically analyzed to highlight the comparative strengths and limitations of each therapeutic option.

Results

Study Selection Process

**Figure 1:**
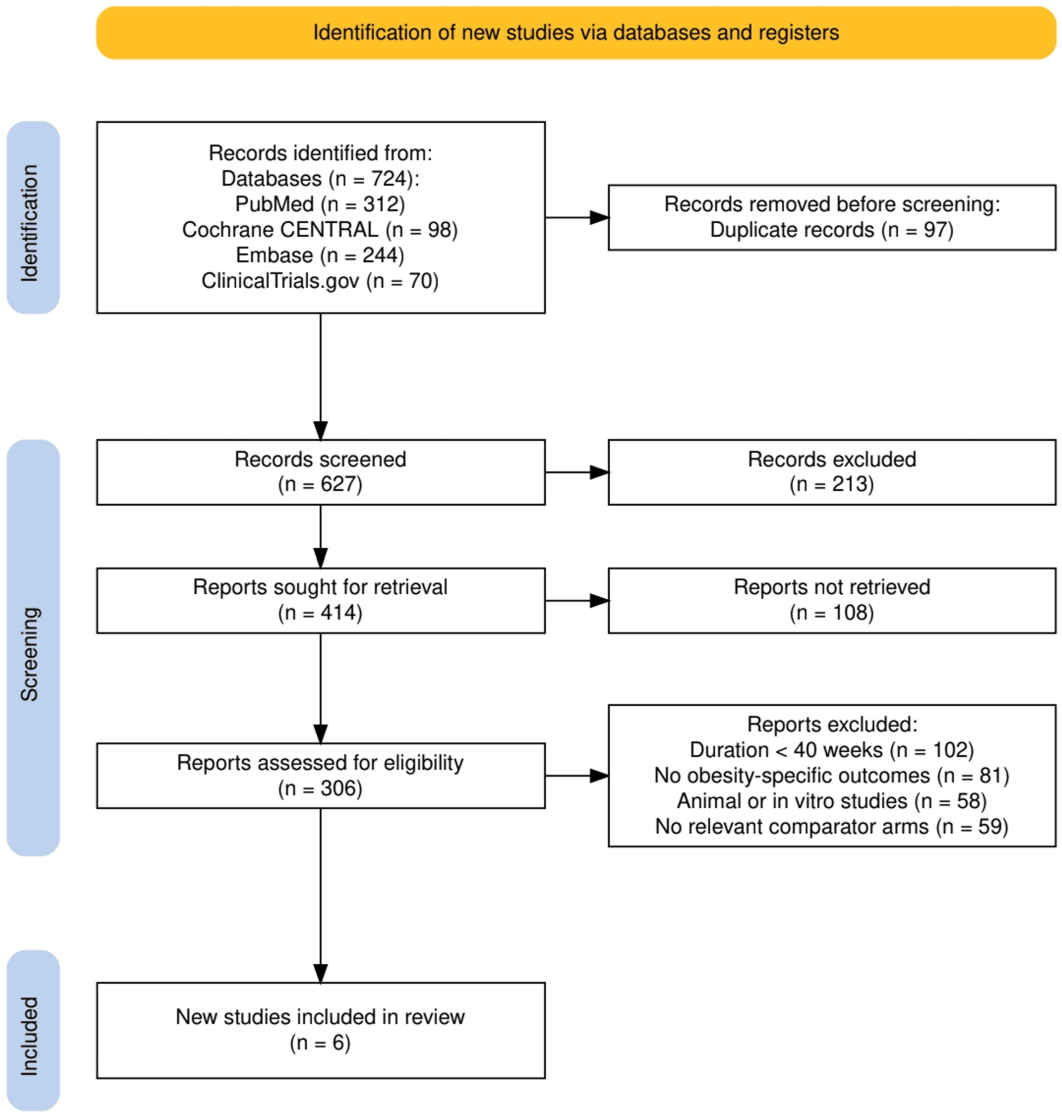
The PRISMA flowchart represents the study selection process. PRISMA: Preferred Reporting Items for Systematic reviews and Meta-Analyses

The study selection process followed PRISMA 2020 guidelines and is illustrated in Figure 1. A total of 724 records were identified through comprehensive searches of PubMed (n=312), Cochrane CENTRAL (n=98), Embase (n=244), and ClinicalTrials.gov (n=70). After the removal of 97 duplicate entries, 627 records remained for screening. Of these, 213 records were excluded based on title and abstract review for reasons such as irrelevance or failing to meet preliminary criteria. The full texts of 414 studies were sought for retrieval, of which 108 could not be accessed. The remaining 306 reports were assessed for eligibility. Ultimately, 300 were excluded for the following reasons: duration of intervention less than 40 weeks (n=102), absence of obesity-specific outcomes (n=81), use of animal or in vitro models (n=58), and lack of relevant comparator arms (n=59). Six studies met all inclusion criteria and were included in the final systematic review.

Characteristics of the Selected Studies

As shown in Table 1, the six selected studies varied in population demographics, GLP-1 receptor agonists used, and follow-up durations; yet, they consistently demonstrated sustained weight loss and acceptable safety profiles. Interventions included semaglutide, liraglutide, tirzepatide, and exenatide, with treatment durations ranging from 40 to 120 weeks. Across adult and adolescent populations, both with and without comorbidities such as type 2 diabetes or cardiovascular disease, GLP-1 RAs have led to notable reductions in body weight and improvements in metabolic parameters. Some studies also highlighted the added benefits of adjunct lifestyle interventions such as exercise. Safety profiles were largely favorable, though gastrointestinal side effects and discontinuation rates varied by agent and dose. Collectively, the studies reinforce the long-term therapeutic potential of GLP-1 RAs in obesity management and underline the importance of sustained treatment strategies.

**Table 1:** Summary of randomized controlled trials evaluating long-term efficacy and safety of GLP-1 receptor agonists for obesity management. GLP-1 RA: glucagon-like peptide-1 receptor agonist; BMI: body mass index; T2D: type 2 diabetes; SAEs: serious adverse events; CVD: cardiovascular disease; RCT: randomized controlled trial; GI: gastrointestinal

References	Population	Intervention	Comparison	Primary outcomes	Follow-up duration	Key findings (efficacy)	Key findings (safety)	Comments/notes
Wilding et al., 2022 [10]	N=327; adults with BMI ≥30 or ≥27 with ≥1 comorbidity; no diabetes	Semaglutide 2.4 mg weekly for 68 weeks, followed by 52-week withdrawal	Placebo + lifestyle; all treatments stopped at 68 weeks	Weight regain, cardiometabolic parameters post-treatment	120 weeks (68 weeks treatment + 52 weeks off-treatment)	Participants regained ~2/3 of weight lost post-treatment; net weight loss at 120 weeks: 5.6%	No new safety signals reported; exploratory analysis	Confirms obesity as chronic condition requiring ongoing pharmacotherapy
Lundgren et al., 2021 [11]	N=195; adults with BMI 32-43; no diabetes; post 8-week low-calorie diet	Liraglutide 3.0 mg/day for 52 weeks±exercise	Placebo±exercise (4-arm RCT)	Change in body weight; secondary: body-fat %, metabolic health	52 weeks	Greatest weight/fat loss with liraglutide + exercise; liraglutide alone >placebo	Increased HR and gallstones with liraglutide; better tolerance in combo group	Demonstrates synergistic effect of liraglutide and exercise in weight maintenance
Kushner et al., 2025 [12]	Patients with overweight/obesity and established CVD	Semaglutide 2.4 mg weekly; long-term (SELECT study)	Placebo	SAEs, discontinuations, GI effects, suicide risk	Several years (exact not specified)	Efficacy not reported; focus on safety; fewer SAEs with semaglutide	Higher discontinuation due to GI events; no new safety concerns	Largest long-term safety trial of semaglutide; confirms tolerability
Bergman et al., 2025 [13]	N=6,246; adults with T2D; overweight/obese; SURPASS 1-5 trials	Tirzepatide 5/10/15 mg weekly for 40-52 weeks	Placebo, semaglutide 1 mg, insulin degludec, insulin glargine	Time in glycemic control and ≥5% weight reduction	40-52 weeks	Tirzepatide led to more sustained weight and glycemic control vs all comparators	Efficacy-focused post hoc	Post hoc, supports durability of tirzepatide outcomes
Jensen et al., 2022 [14]	N=130 (completed); adults with obesity; post low-calorie diet	Liraglutide 3.0 mg/day for 1 year±exercise	Placebo±exercise (4-arm RCT)	Appetite, sedentary time, eating behavior	52 weeks	Liraglutide prevented appetite decline; combo improved cognitive restraint and sedentary time	No serious safety issues noted	Behavioral extension of Lundgren 2021; supports liraglutide's role in behavioral modification
Bensignor et al., 2024 [15]	N=66; adolescents (mean age 16 years) with severe obesity	Exenatide extended-release, once weekly for 52 weeks	Placebo	BMI% change; predictors like leptin, eating behavior	52 weeks	Lower leptin response predicted better weight maintenance with exenatide	No major safety issues reported	Adolescent-focused; novel look at hormonal predictors

Quality Assessment

Table 2 summarizes the risk of bias assessment across the included studies using the Cochrane Risk of Bias 2.0 tool. Most trials demonstrated a low risk of bias in key domains, such as randomization, adherence to intended interventions, outcome measurement, and result reporting. However, some studies presented concerns due to factors such as post hoc analysis design, missing outcome data, or reliance on indirect measures for certain endpoints. Despite these issues, the overall quality of evidence was considered high, reinforcing confidence in the validity and applicability of the findings for long-term obesity management using GLP-1 receptor agonists.

**Table 2:** Risk of bias assessment of included trials using the Cochrane RoB 2.0 tool. RoB: risk of bias; RCT: randomized controlled trial; GLP-1 RA: glucagon-like peptide-1 receptor agonist

References	Randomization process	Deviations from intended interventions	Missing outcome data	Measurement of outcome	Selection of reported results	Overall risk of bias
Wilding et al., 2022 [10]	Low risk	Low risk	Low risk	Low risk	Low risk	Low risk
Lundgren et al., 2021 [11]	Low risk	Low risk	Low risk	Low risk	Low risk	Low risk
Kushner et al., 2025 [12]	Low risk	Low risk	Some concerns	Low risk	Low risk	Some concerns
Bergman et al., 2025 [13]	Some concerns (post hoc)	Low risk	Low risk	Low risk	Some concerns	Some concerns
Jensen et al., 2022 [14]	Low risk	Low risk	Low risk	Low risk	Low risk	Low risk
Bensignor et al., 2024 [15]	Some concerns	Low risk	Low risk	Some concerns (indirect measures)	Low risk	Some concerns

Discussion

Our systematic review demonstrates that GLP-1 receptor agonists (GLP-1 RAs), including semaglutide, liraglutide, tirzepatide, and exenatide, offer sustained efficacy in weight management, with consistent improvements in glycemic control among patients with obesity or overweight. Semaglutide 2.4 mg weekly showed an average weight loss of 17.3% at 68 weeks, though two-thirds of this loss was regained after withdrawal, reinforcing the need for continued therapy [10]. Tirzepatide, evaluated in the pooled SURPASS trials, maintained superior glycemic control and ≥5% weight loss at 40-52 weeks in a population with type 2 diabetes, outperforming semaglutide and insulin comparators [13]. Liraglutide 3.0 mg/day, especially when combined with exercise, led to greater reductions in fat mass and improved metabolic health over 52 weeks [11,14]. Notably, adolescents receiving exenatide showed improved weight maintenance, with lower leptin responses predicting better outcomes [15]. Across studies, gastrointestinal adverse events were the most common safety concern, particularly with semaglutide and liraglutide, yet no new long-term safety signals emerged. Overall, tirzepatide demonstrated the most durable dual benefits (weight and glycemic control), while liraglutide showed added behavioral effects when combined with lifestyle interventions.

The sustained efficacy of GLP-1 RAs supports a paradigm shift in the clinical management of obesity, moving from short-term weight loss strategies to long-term pharmacotherapy for chronic disease control [16]. Unlike traditional interventions such as low-calorie diets or behavioral counseling, which often result in ≥30% weight regain within one year, pharmacologic agents like semaglutide and tirzepatide have demonstrated durable weight reductions of 10-20%, alongside improvements in cardiometabolic risk factors, such as blood pressure, HbA1c, and lipid profiles. Additionally, liraglutide has shown positive behavioral effects, such as enhanced cognitive restraint and reduced sedentary time, aligning with a more holistic model of obesity treatment [14]. These agents not only address excess weight but also reduce the risk of obesity-related comorbidities, such as type 2 diabetes and cardiovascular disease. Clinically, this reinforces the need to reframe obesity as a chronic, relapsing condition that demands sustained pharmacological intervention in the same manner as hypertension or hyperlipidemia, rather than as a condition treatable with short-term or lifestyle-only solutions [17].

GLP-1 receptor agonists (GLP-1 RAs) exert long-term effects on weight and metabolic regulation through a complex interplay of hormonal, neural, and behavioral mechanisms [18]. By mimicking endogenous GLP-1, these agents stimulate insulin secretion, inhibit glucagon release, and delay gastric emptying, leading to enhanced satiety and reduced food intake. Beyond these effects, they modulate central appetite-regulating pathways, particularly in the hypothalamus and brainstem, reducing reward-driven eating behaviors. Behavioral adaptations such as increased cognitive restraint and reduced sedentary time were particularly notable in studies involving liraglutide combined with exercise [14]. Additionally, findings from adolescent trials suggest that individual hormonal profiles, such as baseline leptin responsiveness, may influence treatment success, highlighting the potential for biologically guided personalization in the future [15].

Although GLP-1 RAs are generally well tolerated, gastrointestinal adverse effects - including nausea, vomiting, and diarrhea - remain the most common side effects, leading to higher discontinuation rates, particularly in early treatment phases [19]. Liraglutide was also associated with a modest increase in gallstone incidence, especially with rapid weight loss [11]. Importantly, combination therapies and gradual dose escalation have been shown to improve tolerability, suggesting practical strategies for enhancing adherence. From a public health perspective, while GLP-1 RAs present higher upfront costs, they may prove cost-effective over time by reducing obesity-related complications and healthcare utilization [20]. However, accessibility remains a barrier, especially in low- and middle-income settings, and continuous, possibly lifelong treatment may be necessary to sustain benefits, raising questions about health system sustainability and equitable implementation [21].

This review's strengths include the inclusion of multiple recent, high-powered RCTs, several with sample sizes exceeding 1,000 participants and follow-up periods up to two years. Studies captured a diverse range of populations, including adolescents and patients with comorbid conditions such as type 2 diabetes and cardiovascular disease. Furthermore, secondary outcomes such as appetite regulation, behavioral changes, and hormonal predictors add depth to the evidence. However, notable limitations include heterogeneity in study designs, treatment durations, comparator arms, and outcome reporting, making direct comparisons challenging. Additionally, while some data extend to 120 weeks, real-world evidence on treatment adherence, long-term safety, and durability beyond two years is still lacking. Reliance on post hoc analyses in certain studies also limits the strength of causal inferences.

Despite promising evidence, several important research gaps remain. Head-to-head trials directly comparing GLP-1 RAs (e.g., semaglutide vs. tirzepatide or exenatide) are scarce and necessary to inform optimal agent selection. Furthermore, underrepresented populations - including adolescents, the elderly, and individuals with psychiatric or eating disorders - require focused study. There is also a critical need for long-term (>2 years) outcome data, especially regarding sustained weight loss, cardiovascular event reduction, and all-cause mortality. Lastly, emerging insights into biomarkers such as leptin and satiety hormone responses should prompt trials investigating personalized or stratified approaches to GLP-1 RA therapy to maximize efficacy and minimize discontinuation [22].

## Conclusions

This systematic review demonstrates that GLP-1 receptor agonists - particularly semaglutide, liraglutide, and tirzepatide - are effective and generally well-tolerated options for achieving and maintaining long-term weight loss in individuals with obesity. Their benefits extend beyond weight reduction, encompassing improved glycemic control and favorable behavioral and metabolic changes. However, the sustainability of these outcomes often depends on continued treatment, reinforcing the view of obesity as a chronic disease requiring long-term pharmacological management. While current evidence is promising, especially from recent high-quality trials, further research is needed to refine patient selection, optimize treatment duration, and ensure accessibility in diverse clinical settings. These findings support the integration of GLP-1 RAs into comprehensive, individualized obesity management strategies.
